# Progressive Indosinian N-S Deformation of the Jiaochang Structure in the Songpan-Ganzi Fold-Belt, Western China

**DOI:** 10.1371/journal.pone.0076732

**Published:** 2013-10-18

**Authors:** Bin Deng, Shugen Liu, Sun Liu, Luba Jansa, Zhiwu Li, Yong Zhong

**Affiliations:** 1 State Key Laboratory of Oil and Gas Reservoir Geology and Exploitation/Chengdu University of Technology, Chengdu, China; 2 Dalhousie University, Halifax, and Geological Survey of Canada–Atlantic, Dartmouth, Canada; 3 CCDE Geophysical Prospecting Company, Chengdu, China; ETH, Switzerland

## Abstract

Integrated field data, microstructural and three-dimensional strain analyses are used to document coaxial N-S shortening and southward increase in deformation intensity and metamorphism at the Jiaochang structure. Two episodes of deformation (D_1_,D_2_) with localized post-D_2_ deformation have been identified in the area. The first deformation (D_1_) episode is defined by a main axial-plane of parallel folds observable on a micro- to kilometer-scale, while the second episode of deformation (D_2_) is defined by micro-scale metamorphic folds, associated with E–W oriented stretching lineation. These processes are the result of Indosinian tectonism (Late Triassic to Early Jurassic) characterized by nearly coaxial N-S compression and deformation. This is indicated by E–W trending, sub-parallel to parallel foliation (S_1_, e.g. axial-plane of folds, and S_2_, i.e. axial-plane of metamorphic folds, crenulation cleavage) and lineation (L_1_, e.g. axis of folds, and L_2_, i.e. stretching lineation, axis of metamorphic folds and B-axis of echelon lens). Most of the porphyroblasts and minerals (e.g. pyrite, biotite) show two growth phases with localized growth in the third phase (muscovite). The progressive D_1_–D_2_ structure is widespread in the south of the Jiaochang area, but only D_1_ structure crops out at the north. The strain intensity (γ), compression ratios (c%) and octahedral strain intensity (ε_s_) are similar across the Jiaochang structure (i.e., γ ≈ 1.8, c ≈ 27%, ε_s_ = 0.9), showing a broad range of Flinn values (K = 0.77 to 7.57). The long-axis orientations are roughly symmetric between two limbs of the structure. Therefore, we suggest that the architecture of the Jiaochang structure has been controlled by coaxial N-S shortening and deformation (D_1_–D_2_) during the Indosinian tectonic epoch, with insignificant post-D_2_ deformation.

## Introduction

**Figure 1:**
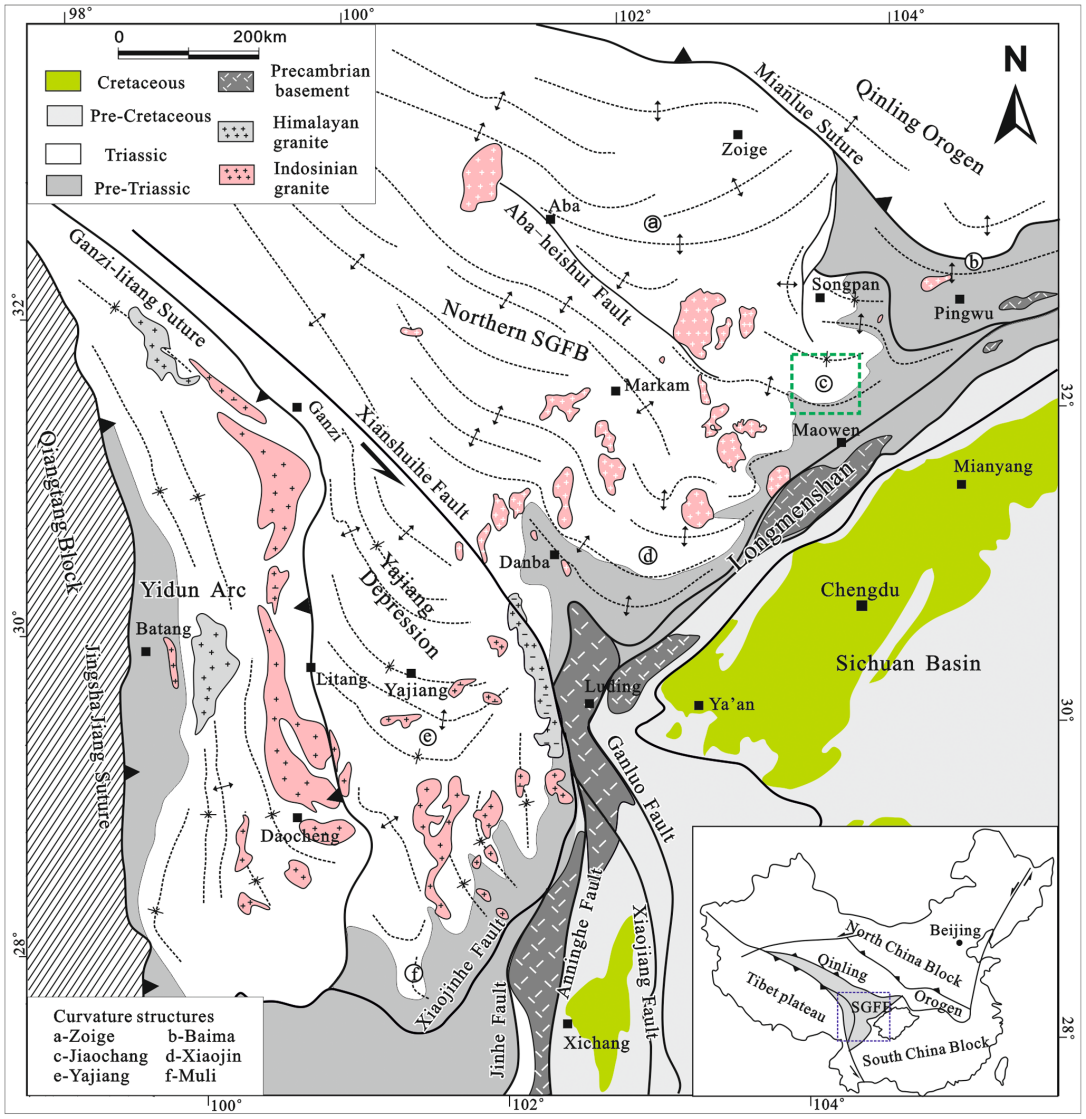
Geological map showing the curvature structures in the Songpan-Ganzi fold-belt. Detailed structural map of the region marked with a green box is presented in Fig. 2.

The oroclinal bending, which is one of the most striking features in the Songpan-Ganzi fold belt, shows an obvious change in the orientation of structure from northwest direction in the west, to E-W direction and northeast direction farther to the east near the border with Longmenshan (*Fig. 1*). It includes Zoige, Baima, Jiaochang, Xiaojin, Yajiang, and Muli structures in the Songpan-Ganzi area. All of these structures are of symmetric and parabolic shape and have parallel trend lines in their interior. These structures were interpreted by several authors [1]–[3] to be related to sinistral transpressive boundary, namely the Maowen-Wenchuan fault between the Songpan-Ganzi fold-belt and Longmenshan.

Wang et al. [2] argued that NE-SW shortening and sinistral shearing took place along the boundary fault to accommodate the structures curvature. Harrowfield and Wilson [3] concluded that the structures are result of two-phase south-oriented deformation with transpressive shortening at the southeastern margin. Most of the authors recognized the significance of sinistral transpression along Maowen-Wenchuan fault [2], [4], [5], however, Xu et al. [6] argued that the N-S and E-W deformation took place along different boundaries. They suggested that the deformation is result of a closure of Qiangtang, North China and South China Blocks, being the dominant processes forming the curvature structures. In contrast Chen et al. [1] attributed the structures to the superimposed deformation by sinistral shortening and early SW-oriented shortening. Unraveling the curvature-producing mechanism and processes is difficult due to the lack of detailed studies of these structures. Various authors noted that different factors could have played roles in the formation of curvature structures [7]–[9], such as basin-controlled mechanism [10] and slipping at the boundary [11].

In this study, we present new observations based on field data, microstructure and three-dimensional strain analyses from the Jiaochang structure at the north Songpan-Ganzi fold-belt. We have recognized two principal episodes of N-S oriented deformation (D_1_,D_2_) at the Jiaochang structure, which mostly account for the architecture of the Jiaochang area.

## Geologic Setting

The Songpan-Ganzi Fold-Belt (SGFB) is a triangular area with widespread Triassic flysch located at the eastern margin of the Tibetan Plateau (Fig. 1). It is bounded to the south, southeast, north and northwest by the Qiangtang, South China, North China and Qiadam blocks. The fold belt was formed during Late Triassic by multiphase continent-continent collision between the Qiangtang, North China and South China blocks [6], [12]–[13]. The SGFB experienced south-oriented shortening and crustal thickening [3], [6], which produced upright and isoclinal folds, with the strata intruded by undeformed, crosscutting granitic plutons during Late Triassic time [14], [15]. These granites have been interpreted to be the consequence of partial melting of continental crust [14], [15]. In the northern part of the Songpan-Ganzi area, the Triassic flysch is unconformably overlain by weakly deformed, unmetamorphosed Jurassic red beds. The nearly horizontal inclination and weak deformation of Jurassic-Cretaceous strata in the Songpan-Ganzi area indicate lack of significant tectonic deformation post Triassic time.

The widespread Songpan-Ganzi flysch is comprised by 5∼15 km of mudstone and shale, exposed to low- to medium-grade greenschist-facies metamorphism [16]. Flysch is underlain by Sinian-Paleozoic sequences comprised by limestones and clastics, which experienced higher-grade medium-pressure metamorphism [5], [17]. A large-scale decollement has been recognized at the southern and eastern part of the Songpan-Ganzi area [3], [6], where it separates the Triassic sequence from the underlain strata. The strong south-oriented compression and shortening has an important influence on tectonic deformation of the region during Late Triassic time [3], [18]–[19]. Afterwards, the SGFB has experienced a very long period of thermal and tectonic quiescence through the Middle Jurassic to Late Cretaceous [13], [20]. During the Himalayan collision between India and Asia, the NW-striking strike-slip faults and thrusts strongly modified the SGFB [2], [12]. Various structures were reactivated at the same time, including NE-trending Longmenshan, which is still active today (e.g., the 1933 *M*_w_ 7.3 Diexi and 1976 *M*_w_ 6.9 Songpan earthquakes).

**Figure 2:**
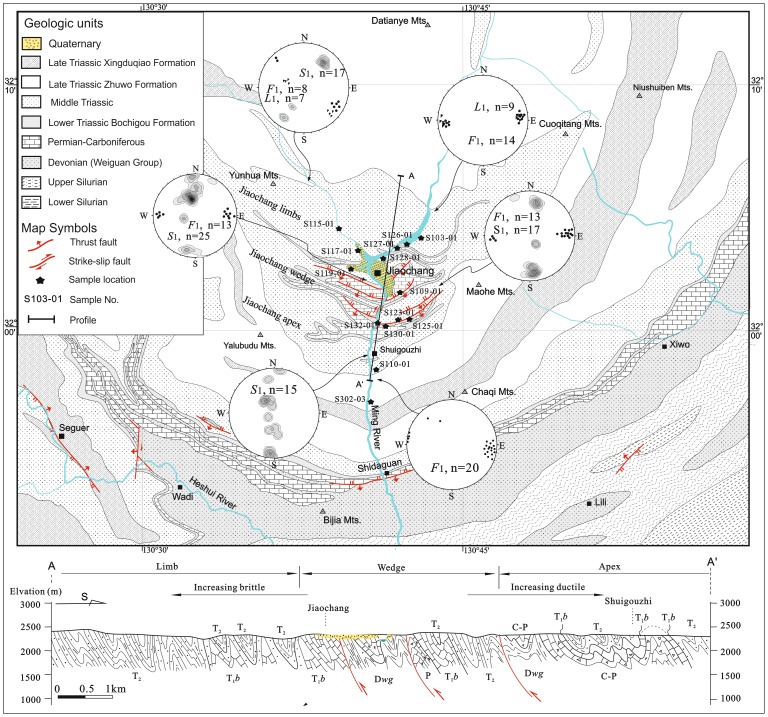
Geologic and structure map of the Jiaochang area in the Songpan-Ganzi fold-belt. All stereonets are lower-hemisphere equal area projection, showing the S1 foliation. The solid circles and triangles in the stereonets stand for F1 hinge and L1 intersection lineation, respectively.

Our study area in the Jiaochang structure is located at a transition region between the northern SGFB and the Maowen-Wenchuan ductile shear zone in the Longmenshan. It is characteristic by the occurrence of symmetric, parallel and parabolic structures (*Fig. 2*). There crop out widespread Mid-to-late Triassic metamorphic sandstone and slate, and Lower Triassic marble. Upper Paleozoic marble locally outcrops at the south of the Jiaochang area. Jiaochang structure is cut by three, E-W striking, south-dipping thrust faults, and one N-S striking sinistral fault along the center of the structure. Furthermore, it is dissected by the duplex thrusts, which allows roughly subdivide the Jiaochang structure into three parts, which from the south to the north are: the apex, the wedge and the limbs.

## Structural Geometry of the Jiaochang Area

### 3.1 The Southern Region: The Jiaochang Apex

**Figure 3:**
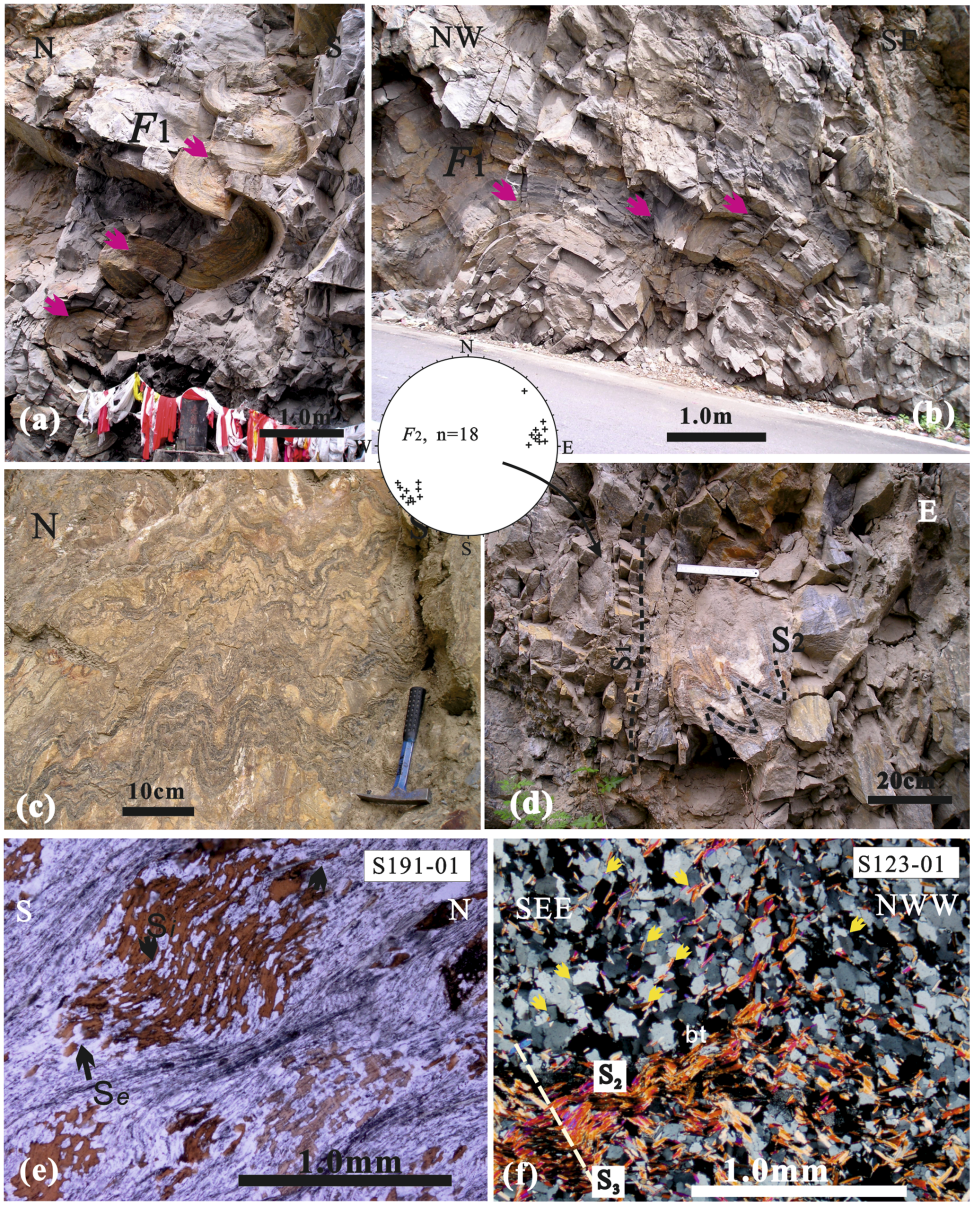
Field photographs and photomicrographs from the southern region of the Jiaochang structure. a–b) D_1_ E–W striking, parallel folds of centimeter to meters scale in the Middle Triassic strata. c) metamorphic polyharmonic -folds (S0//S1) in the Middle Triassic strata. d) Sub-parallel multiphase foliations (S_1_ and S_2_) and the second lineation (F_2_). The stereonet shows NE∼NEE trending F_2_ lineation. e) Rotated helicitic structure (porphyroblast) of biotite in lepidoblastic quartz-mica schist. The quartz inclusions (S_i_) rotated about 75° with respect to matrix minerals (S_e_) during growth, indicating progressive deformation and metamorphism. f) Grain Boundary Migration (GBM) recrystallization in lepidoblastic to equigranular mica-quartz schist. The micas (pervasive metamorphic foliation S_2_) pin some grain boundaries in quartz minerals and were later crenulated to form crenulation cleavage (S_3_).

The Jiaochang structure is dominated by nearly E-W trending, upright-inclined F_1_ fold (*Fig. 2*). At the southern region, Devonian Weiguan Group to Middle Triassic sequences were deformed by parallel-to-similar fold from centimeter to meters in scale (*Fig. 3a*, *Fig. 3b*). Such structure is typical of the first episode of deformation (D_1_) across the Songpan-Ganzi Fold-Belt, as described by Dirk et al. [4], Worley and Wilson [5] and Harrowfield and Wilson [3].

Polyharmonic fold (*Fig. 3c*) and multiphase foliation (*Fig. 3d*) seen in the outcrops is a typical (D_2_) deformation accompanied by metamorphism in the southern region. It indicates medium to high intensity of deformation and metamorphism. Immediately south of Shuigouzhi along the Minjiang River, deformation of the upper Paleozoic strata, particularly of the marble rock, is marked by a distinct metamorphic fold (S_2_ and F_2_) (*Fig. 3c* *,* *Fig. 3d*). The F_2_ axis has a NE∼NEE trend with low-angle plunge. It is sub-parallel to F_1_ axis (*Fig. 2*), indicative of coaxial deformation (D_1_ and D_2_).

Few of the pervasive foliation (e.g. axial cleavage, crenulation cleavage) is preserved. The original sedimentary bedding is only preserved in metamorphic porphyroblasts (*Fig. 3e*). Microstructure in mica-quartz schist shows evidence of quartz recrystallization, dominantly through grain boundary migration (GBM). The new quartz grains with smaller subgrains have interlobate to ameboid grain boundaries (*Fig. 3f*), which is indicative of GBM recrystallization at higher temperatures of ca. 500∼600°C [21]. Syntectonic rotated helicitic structures (*Fig. 3e*) are widespread on micro-scale. The continuity between quartz inclusion (S_i_) and matrix mineral (S_e_) is marked by opaque-poor bands (i.e. quartz with elongated inclusions) continuous inside and outside of the porphyroblast. It is result of progressive deformation (D_1_ and D_2_). Thus we suggest that formation of D_1_ and D_2_ took place at approximately same time, or that the D_2_ was formed slightly later.

Only localized post-D_2_ deformation (D_3_) has been identified in the north of Shuigouzhi, as crenulation cleavage (S_3_) of pervasive metamorphic foliation (S_2_) (*Fig. 3f*).

### 3.2 The Central Region: The Jiaochang Wedge

Further north of Shuigouzhi, the moderate to tight F_1_ fold is still E-W striking with a near-horizontal plunge (*Fig. 2*). However, it is associated with a distinct top-to-the-north thrust in the limb or core, to accommodate two northward thrusting sheets.

**Figure 4:**
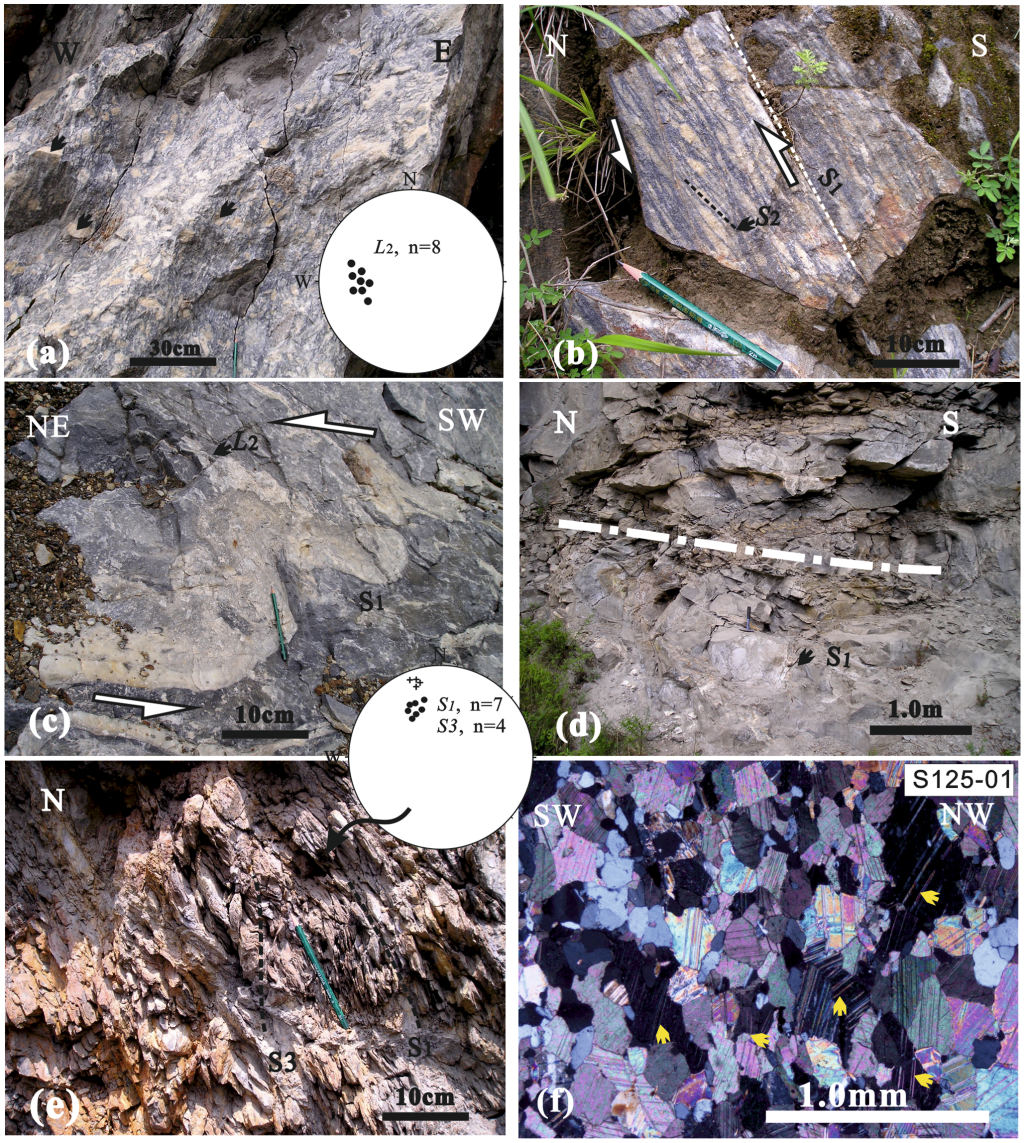
Field photographs and photomicrographs from the central region of the Jiaochang structure. a) A-axis elongated lineation (L_2_) in upper Paleozoic marble. The stereonet shows the westward plunging lineation (elongated calcite) with ∼276°∠36°(trend and plunge). b) Shearing echelon lens with nearly E–W trending B-axis in Middle Triassic marble. c) Sub-parallel bedding veins formed during northward thrusting to accommodate asymmetric fold. Many of these veins are asymmetrically shortened and sheared to form pinch-and-swell structures and are potential kinematic indicators. d) Dragged recumbent fold in Upper Paleozoic with E–W-strike and horizontal plunge. e) Sub-parallel multiphase foliation of the first foliation (S_1_) and later crenulation cleavage (S_3_) in Devonian. The stereonet shows the NNW-ward pole of foliations. f) Growth and deformation twins in calcite with deforming and tapering edges, indicating deformation temperatures of ca.300∼400°C.

In the central region of Jiaochang structure, there are widespread kinematic indicators of north-oriented transport (*Fig. 4b* *,* *Fig. 4c*), some of which are characterized by sinistral shearing (*Fig. 4c*). These structures in conjunction with mineral lineation defined by elongated grains (e.g. calcite (*Fig. 4a*)) and shearing echelon lens (*Fig. 4b*), indicate a consistent D_2_ with ductile deformation. The L_2_ lineation is E-W trending with various plunges, and is sub-parallel to F_1_ axis; the S_2_ foliation is also sub-parallel to S_1_ foliation (*Fig. 4b*). Furthermore, localized D_3_ deformation was identified at the footwall of the thrust fault. The S_3_ crenulated an earlier slaty cleavage, with a very small angle, or being sub-parallel to S_1_ (*Fig. 4e*).

There is a northward decrease in the intensity of deformation and metamorphic grade, from the southern to central region of the Jiaochang. There is widespread growth twin of calcite with deformed and tapered edges (*Fig. 4f*). It indicates lower deformation temperatures (ca.300∼400°C, Passchier and Trouw [21]) than at the southern region.

### 3.3 The North Region: The Jiaochang Limbs

At the north region, the deformation of the Lower- to Middle- Triassic is marked by a distinct change in deformation intensity. The F_1_ fold is also upright, NW to EW striking (*Fig. 2*). Most of the folds have generally axial cleavage (S_1_), defined by an alignment of biotite and elongation of quartz grains. Furthermore, the L_1_ (intersection lineation and bounding axis) is found to be parallel to F_1_ axis with NW to EW strike (*Fig. 2*).

**Figure 5:**
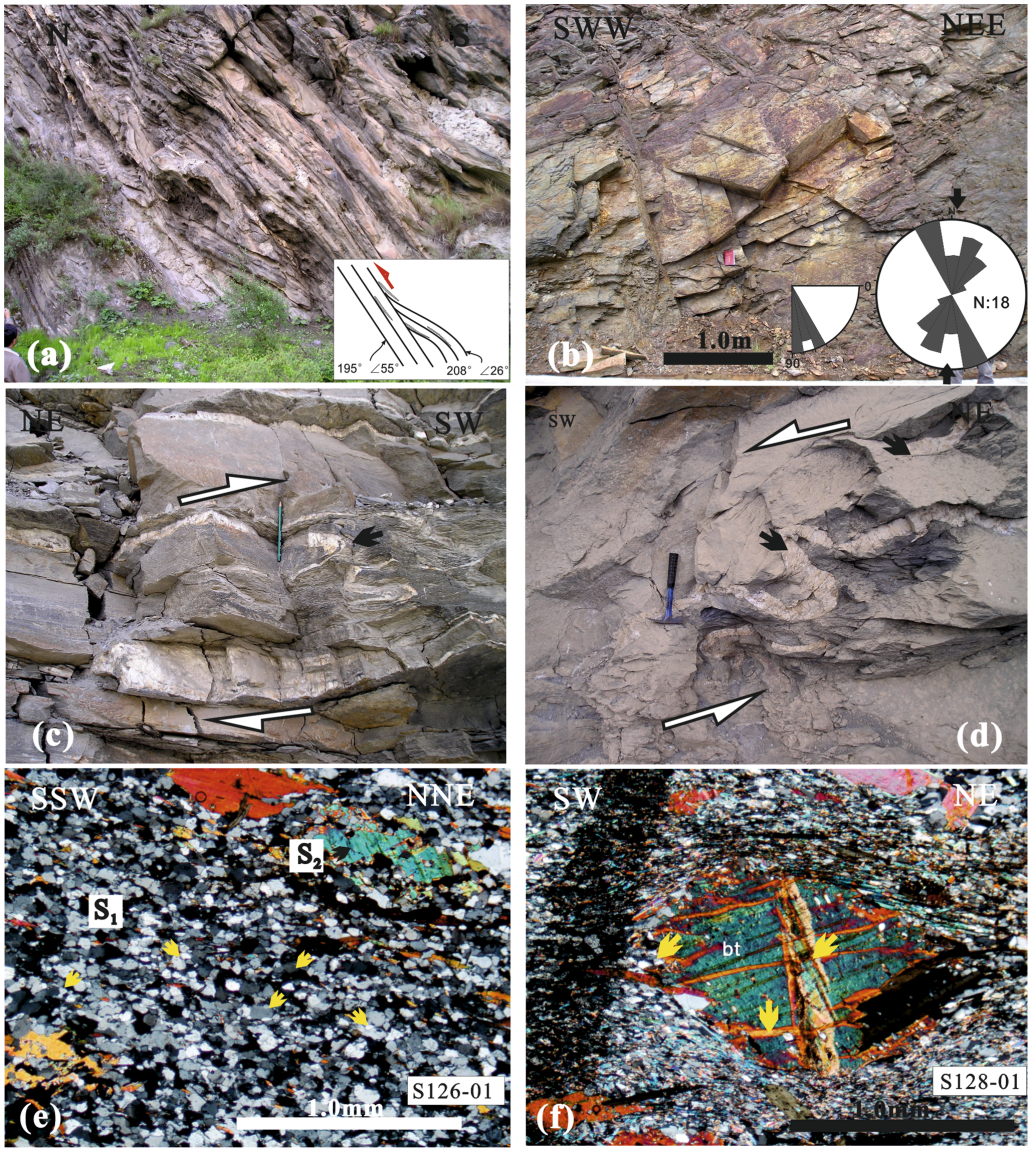
Field photographs and photomicrographs from the northern region of the Jiaochang structure. a) Fault-related fold with E-W-strike and horizontal plunge is result of northward thrusting. b) Conjugated joint. The rose diagram of restored joints shows the latest N–S compression. c) Widespread parallel-layer shearing veins, showing southward thrust and deformation. d) Sub-parallel bedding veins with high-angle plunge, indicating sinistral strike-slip in western limb of the Jiaochang. e) Subgrain Rotation (SGR) recrystallization in equigranular mica-quartz schist, indicating medium deformation temperatures of ca. 400∼500°C. f) Sub-symmetric eye structure (porphyroblast) of biotite in lepidoblastic mica-quartz schist, showing no or little sense of the shear. Biotite grain shows crenulation cleavage and later fracture.

In the direction northward from Jiaochang village towards two limbs, there is widespread occurrence of asymmetric veins, sub-parallel to parallel with bedding, which is result of different shearing. Such veins in the limbs of Jiaochang dominantly show southward, or northward thrust and deformation (*Fig. 5c* *,* *Fig. 5d*). Furthermore, veins in the western limb have much higher plunge than those in the eastern one, indicating sinistral shearing (*Fig. 5d*).

Microstructures in the north region of the Jiaochang structure display evidence of quartz recrystallization through subgrain rotation (SGR) (*Fig. 5e*). This indicates deformation temperatures of ca. 400∼500°C [21], therefore much lower metamorphic and deformation condition than those in the south.

Although no post-D_1_ ductile deformation has been observed, the post-D_1_ brittle deformation is prominent in the northern region. Some of fold limbs display different-scale transporting structure, most of which generally indicates N-oriented thrust, to accommodate fault-related fold (*Fig. 5a*). Moreover, a series of late joints crosscuts the previous structure (*Fig. 5b*). The rose diagram of restored conjugate joint also shows the latest N-S compression.

## Three-Dimensional Strain Analyses

### 4.1 Samples and Methods

The three-dimensional strain analyses in the Jiaochang structure was conducted applying Fry method [22]–[23] on 12 oriented samples. For each sample, the two-dimensional strain was measured across grain-supported area, with approximately 130∼170 grains (mostly 150 grains, both relict and recrystallized grains) in X–Z, Y–Z and X–Y sections, respectively. Then, we constructed three-dimensional strain ellipse based on the two-dimensional strain. It should be noted that recrystallized grains do not yield significant finite strain in metamorphic rocks, thus we measured finite strains with both relict grains and recrystallized grains.

### 4.2 Results

**Figure 6:**
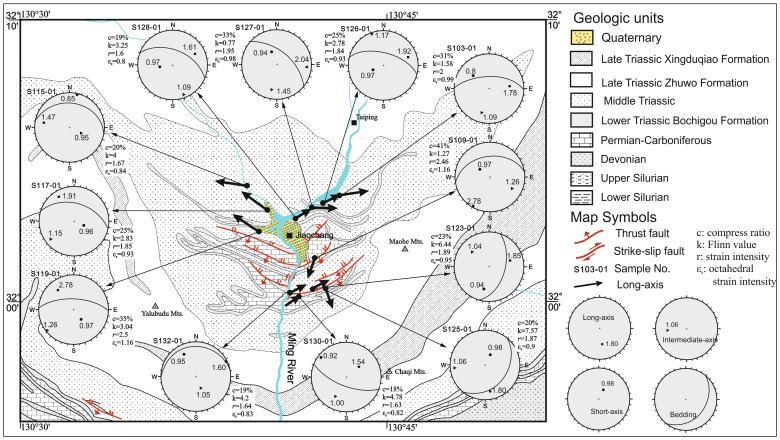
Geological map of the Jiaochang structure showing the major structures and locations with kenematic data. The azimuth of ellipsoid long-axis orientation of the three-dimensional finite strain (bold arrows) indicates the maximume stretching direction and also provides local transport direction. All stereonets are lower-hemisphere equal area projection.

**Figure 7:**
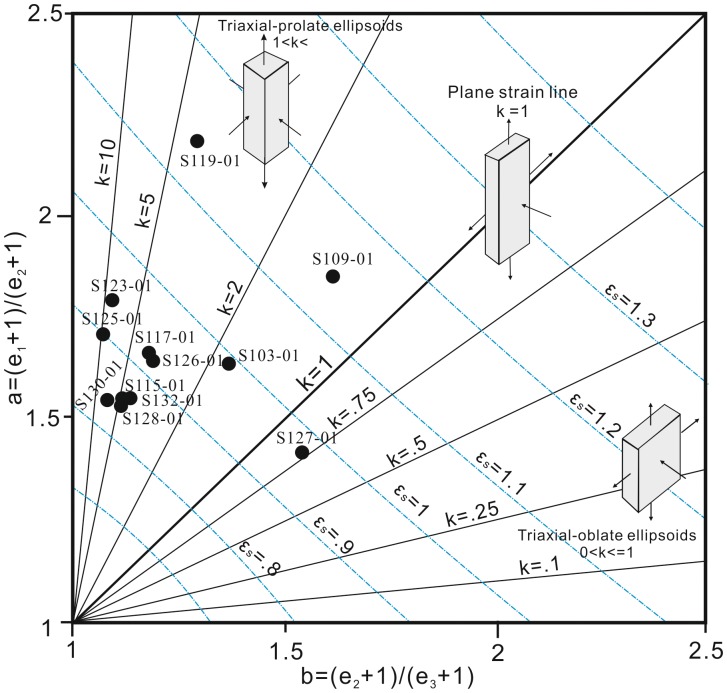
Flinn diagram with contours of Flinn K-values and natural octahedral strain magnitude, ε_s_, from the Jiaochang structure. Note that most samples cluster around K = 5, between ε_s_ = 0.8 to ε_s_ = 1.0.

The detailed results from three-dimensional strain analyses are listed in *Table 1*. The magnitude and orientation of the three principal axis of strain together with bedding orientation are shown in *Fig. 6*. Based on these data, we have constructed Flinn diagram [24] as shown in *Fig. 7* *.*

**Table 1:** Three-dimensional strain data in the Jiaochang structure.

Sample	Principal strains			K	ε_s_	γ	c	e_1_/e_3_	Bedding	Strain orientation			è
	e_1_	e_2_	e_3_							e_1_′	e_2_′	e_3_′	
S115-01	1.47	0.95	0.85	4.00	0.84	1.67	20%	1.73	60°∠193°	25°∠281°	65°∠110°	04°∠12°	11°
S117-01	1.91	1.15	0.96	2.83	0.93	1.85	25%	1.99	42°∠192°	20°∠328°	14°∠233°	66°∠111°	45°
S119-01	2.78	1.26	0.97	3.04	1.16	2.50	35%	2.87	25°∠182°	30°∠323°	02°∠232°	60°∠139°	48°
S103-01	1.78	1.09	0.8	1.58	0.99	2.00	31%	2.22	30°∠180°	38°∠81°	29°∠196°	39°∠312°	36°
S126-01	1.92	1.17	0.97	2.78	0.93	1.84	25%	1.98	31°∠195°	24°∠73°	02°∠342°	66°∠248°	37°
S127-01	2.04	1.45	0.94	0.77	0.98	1.95	33%	2.17	30°∠220°	15°∠94°	26°∠191°	59°∠338°	31°
S128-01	1.61	1.09	0.97	3.25	0.80	1.60	19%	1.66	36°∠202°	28°∠64°	09°∠159°	60°∠266°	45°
S132-01	1.60	1.05	0.95	4.2	0.83	1.64	19%	1.69	76°∠182°	02°∠64°	62°∠158°	28°∠332°	28°
S109-01	2.85	1.54	0.95	1.27	1.16	2.46	41%	3.00	50°∠172°	04°∠210°	30°∠118°	60°∠307°	15°
S123-01	1.85	1.04	0.94	6.44	0.95	1.89	23%	1.97	38°∠286°	36°∠74°	36°∠312°	34°∠193°	46°
S125-01	1.80	1.06	0.98	7.57	0.9	1.78	20%	1.84	54°∠309°	22°∠169°	14°∠265°	63°∠26°	53°
S130-01	1.54	1.00	0.92	4.78	0.82	1.63	18%	1.67	21°∠221°	51°∠51°	37°∠211°	10°∠308°	71°

*Notes*: e_1_,e_2_, e_3_ represent the relative magnitude of principal strains; K-Flinn value; ε_s_ –magnitude of natural octahedral strain; γ –strain intensity; c–compression ratio; e_1_/e_3_-axial ratio of three-dimensional strain ellipsoid; Bedding-sample’s bedding orientation; e_1_′, e_2_′, e_3_′ represent the orientations of principal axis in geographic reference frame; è is the intersection angle between the orientation of the principal axis and the sample bedding.

Strain analyses revealed much higher elongation and moderate octahedral shear. The elongation values range from e_1_/e_3_ = 1.66 to e_1_/e_3_ = 3.00, and octahedral strain values range from ε_s_ = 0.82 to ε_s_ = 1.16, corresponding to a range of strain intensity values from γ = 1.63 to γ = 2.50. In addition, the compression ratios vary from c = 18% to c = 41%, and the Flinn values K change significantly within a range of 0.77∼7.57.

In the northern region of the Jiaochang structure, there is roughly symmetrical orientation of the long-axis, nearly NW to EW trending. The strain intensity, compression ratios and octahedral strain intensity are very similar across the eastern limb. However, these values show a general increase from the north to the south at the western limb (*Fig. 6*), i.e. the intensity values and octahedral strain values increase form γ = 1.67 to γ = 2.5 and from ε_s_ = 0.84 to ε_s_ = 1.16.

Five samples from the central and southern region of the Jiaochang were analyzed. No distinct change has been observed in strain intensity (γ), compression ratios (c%) and octahedral strain intensity (ε_s_) from the north to the south. The values range from γ = 1.6 to γ = 2.4, from c% = 18% to c% = 41%, and from ε_s_ = 0.82 to ε_s_ = 1.16, respectively. However, the Flinn values, with a range of ∼4–7, are larger than those in the northern part. The long-axis of three samples is NE striking, and roughly parallel to the fold axis. However, there are two samples (S109-01 and S125-01) with nearly N-S striking long-axis; both of them sampled from locations within meters from the fault. Thus, we suggest that thrusting deformation of the faults could account for significant change of the long-axis of our oriented samples.

In the Flinn diagram most samples cluster around K = 5 within a range of 0.77∼7.57, as a triaxial-prolate ellipsoid (*Fig. 7*). There is only one sample with K less than 1 (S127-01, K = 0.77). The grain shapes yielded relatively moderate octahedral shear strain shown by a range of strain values from ε_s_ = 0.80 to ε_s_ = 1.16. The Flinn diagram shows that most samples have a pattern of triaxial prolate deformation, suggesting that L tectonite instead of S tectonite is more likely to develop in this area. On the contrary, most of the macro- to micro- structure show well developed S tectonite rather than L tectonite (see detailed discussion in Section 5.2).

## Discussions

### 5.1 Progressive Indosinian D_1_–D_2_ Deformation under N-S Shortening

Our observation supports progressive D_1_–D_2_ tectonic deformation, with insignificant post-D_2_ deformation occurring in the Jiaochang area. The deformation is characterized by nearly coaxial N-S compression, indicated by sub-parallel to parallel foliations (S_1_,S_2_) and lineations (L_1_,L_2_).

**Figure 8:**
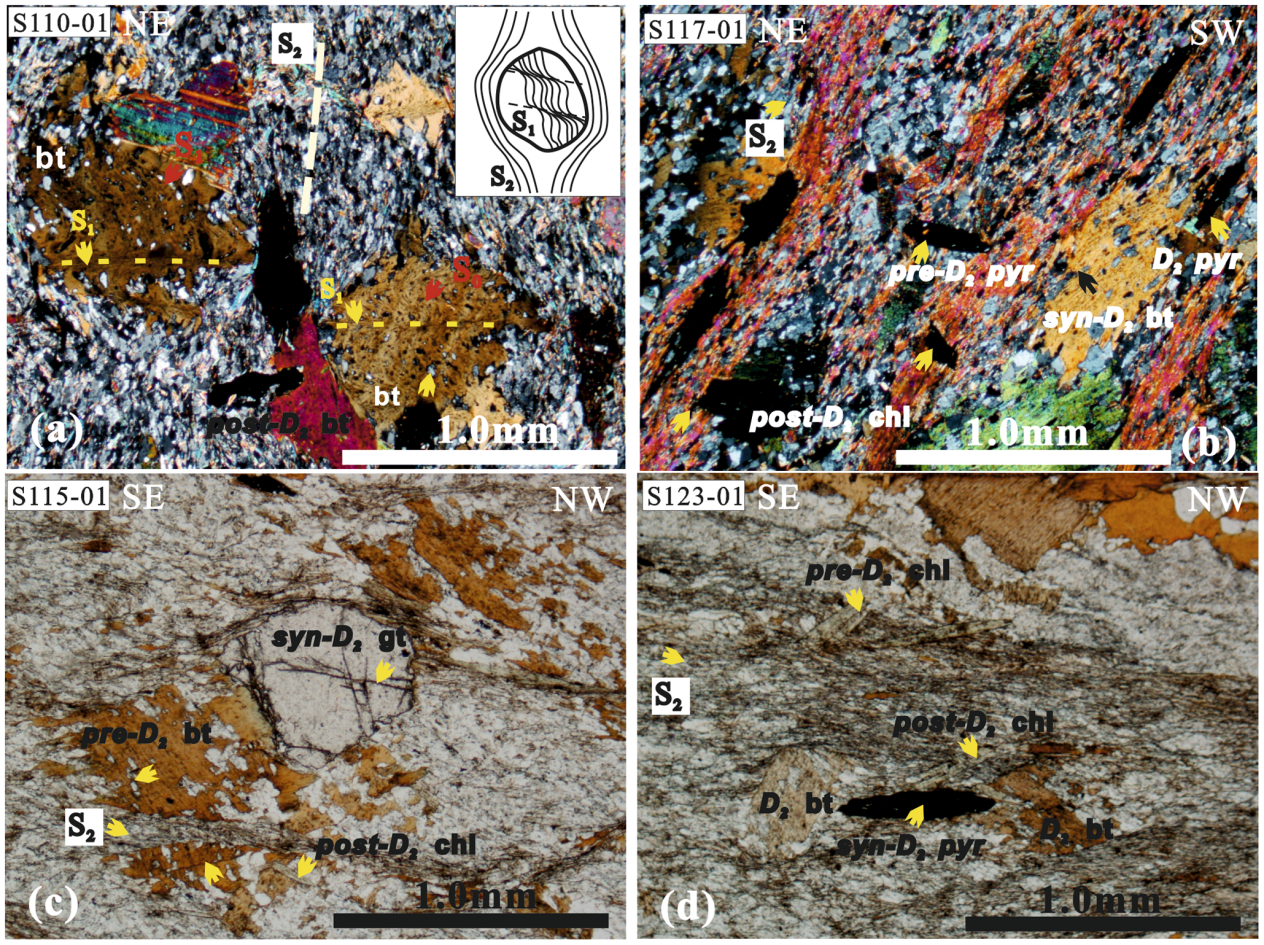
Photomicrographs of metamorphic minerals and of multiphase foliation in the Jiaochang area. a) Biotite porphyroblasts in lepidoblastic quartz-mica schist, showing pervasive schistosity (S_2_) and crenulation cleavage (S_1_). Note that the quartz inclusion (S_0_) is weakly oriented with no or little elongation, which is different than the early deformation (D_1_). The inclusion-poor zones in biotite porphyroblast represent the differentiated limbs of microfolds, showing that the crenulation cleavage in the early matrix has been destroyed since the porphyroblast formed. Biotite porphyroblast shows undulose extinction and is wrapped by deflected matrix mineral in contrast to none-deflection of wrapping mineral (post-D_2_). b) Multiphase growth of biotite, pyrite and chlorite porphyroblasts. Pre-D_2_ porphyroblast is wrapped by deflected matrix mineral in contrast to post-D_2_ porphyroblast. c) Multiphase growth of biotite, garnet and chlorite porphyroblasts. Post-D_2_ chlorite without strain shadow and deflection of wrapping mineral superposes early undulose extinction biotite (pre-D_2_). The elongated inclusion (S_1_) is not continuous with matrix minerals (S_2_, metamorphic foliation of muscovite and biotite) outside of the porphyroblast. Note that the garnet was superposed by late brittle fracture (conjugated joint). d) Multiphase growth of biotite, pyrite and chlorite porphyroblasts. S_2_ shows clear deviation around the metamorphic crystal (syn-D_2_ biotite and pyrite, the latter has local strain-shadow) in which the inclusion is continuous with matrix minerals. There is difference between the pre-D_2_ and post-D_2_ chlorite minerals, post-D_2_ chlorite without strain-shadow cuts across early pervasive foliation (S_2_). Symbols: (bi) = biotite, (ch) = chlorite, (gt) = garnet, (pyr) = pyrite, (mus) = muscovite.

The microstructures in the Jiaochang are also characterized by multiphase foliation and mineral growth (*Fig. 8a*). In thin sections, the alignment of biotite and elongated quartz grains is pervasive and defines the S_2_ (*Fig. 8a* *,* *Fig. 8b*), which is associated with the growth of other minerals. According to Passchier and Trouw [21], pre-tectonic, syn-tectonic, inter-tectonic and post-tectonic porphyroblasts and minerals have obvious differences in deformation, or growth (i.e., deformed inclusions, strain-shadow, deflected S_e_, and undulose extinction). The early porphyroblasts show undulose extinction and are wrapped by deflected matrix minerals, in contrast to the later porphyroblasts without deflection of wrapping minerals (*Fig. 8c* *,* *Fig. 8d*). The late porphyroblasts (e.g., post-D_2_ chlorite) are always without strain shadow and deflection of wrapping minerals, overprinting the early undulose extinction minerals (*Fig. 8d*). Most syn-D porphyroblasts and minerals have continuity of the inclusions (S_i_) to the matrix minerals (S_e_) (*Fig. 3e* *,* *Fig. 8a*). The long axis generally parallels the pervasive schistosity.

**Figure 9:**
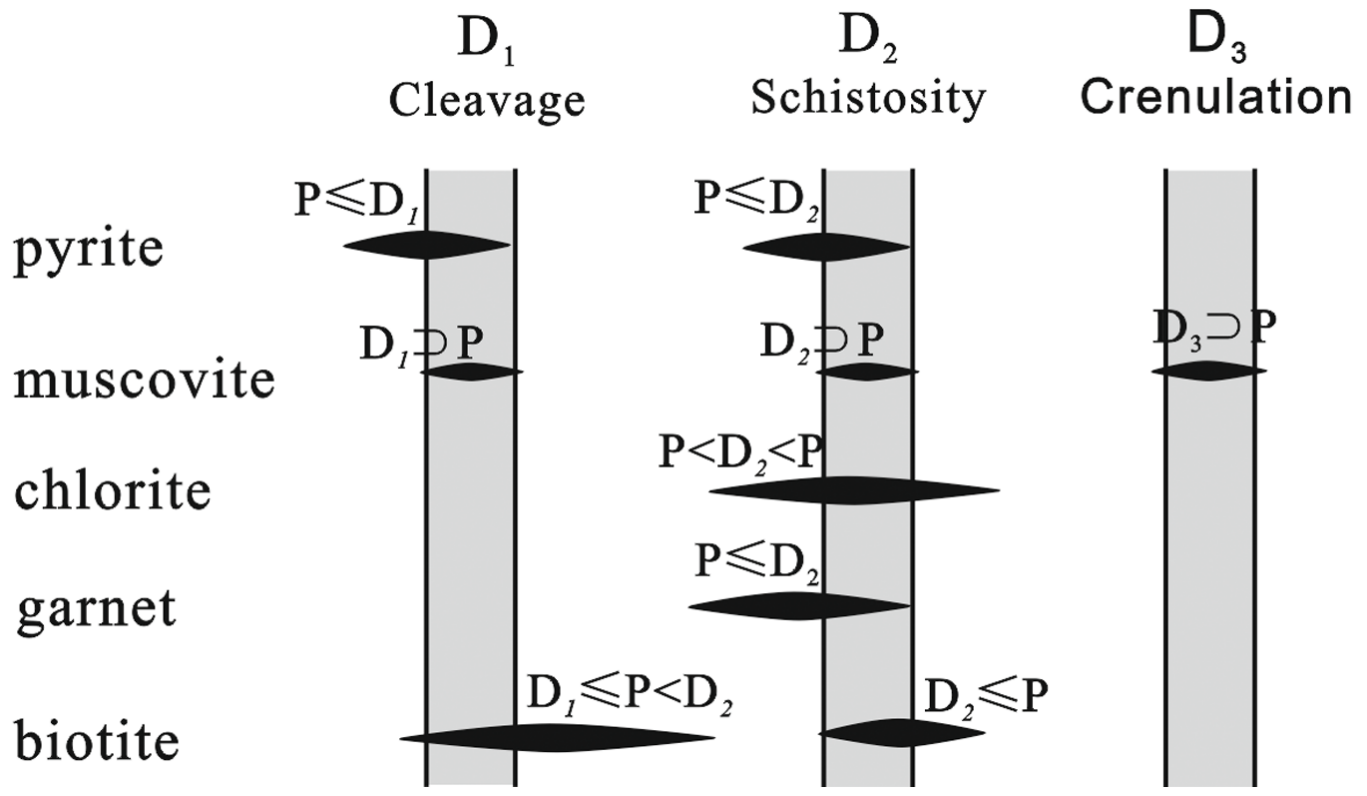
Schematic representation of pre-, inter-, syn, and post-tectonic prophyroblast and mineral growth, in which the D_1_,D_2_ and D_3_ correspond to the multiphase deformations of foliation. Pyrite is divided into pre- to syn-D_1_ and pre- to syn-D_2_. Biotite is divided into syn- to post D_1_, and syn-D_2_. Chlorite and garnet are pre- to post-D_2_, and pre- to syn-D_2_, respectively. Furthermore, muscovite is divided into three growth phases, syn-D_1_, and syn-D_2_ and syn-D_3_.

Therefore, we suggest that the multiphase growth of minerals and porphyroblasts is correlated with the multiphase deformation in the Jiaochang area (*Fig. 9*). In general, most of the minerals grew during the first and second episode of deformation (D_1_ and D_2_); only part of muscovite grew during the third episode of deformation (D_3_). In outcrops, the localized post-D_2_ mineral growth indicates that post-D_2_ deformation had insignificant impact on tectonic architecture of the Jiaochang area. It suggests that the progressive D_1_–D_2_ deformation predominantly accounts for the Jiaochang structure. Furthermore, there are widespread syntectonic structures and recrystallization with grain boundary migration, that makes it difficult to identify which episodes of deformation is more important. Therefore, we argue that the three-dimensional strain should be interpreted with the D_1_–D_2_ progressive process and insignificant post-D_2_ deformation.

In the region, the crosscutting relationship between the folded Late Triassic sequence and the Late Triassic to Early Jurassic (Indosinian) pluton [14], [25], indicates that the architecture of SGFB was shaped by the tectonics during Indosinian orogeny. Thus, progressive D_1_–D_2_ deformation in Jiaochang area is roughly equivalent to the pervasive D_1_ deformation with the superposed D_2_ deformation during Indosinian epoch, as described by Dirks et al. [4], Worley and Wilson [5] and Harrowfield and Wilson [3], and to the “syn-metamorphic” deformation as described by Burchfiel et al. [12].

However, the post-D_2_ deformation (D_3_ and/or D_4_) described by Dirk et al. [4], Chen et al. [1] and Worley and Wilson [5] along the eastern margin of the Songpan-Ganzi area (Longmenshan) does not distinctly crop out in the Jiaochang. As suggested by Harrowfield and Wilson [3], the post-D_2_ deformation is localized in the outcrops in the southern part of the SGFB. In the Jiaochang area, the progressive D_1_–D_2_ structure with ductile deformation is widespread in the south, and the D_1_ structure crops out in the north. These differences show clear heterogeneity in multiphase deformation and metamorphism between the SGFB and Longmenshan, as well as in the SGFB interior. Why such heterogeneous deformation (post-D_2_) is lacking in the Jiaochang structure remains unknown.

### 5.2 Progressive N-S Shortening of the Jiaochang Structure

**Figure 10:**
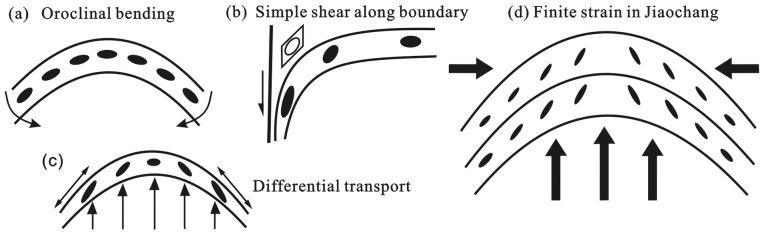
Diagram of strain pattern and mechanism of formation of orogen curvature. a) Oroclinal bending, strain ellipse remains the same but varies in orientation without undergoing tangential extension (after Ries and Shackleton [26]); b) Simple shear along boundary, strike-slip shearing along one limb of orogen curvature causes stretch and deformation increase toward the boundary fault (after Marshak [7]); c) Differential transport, oroclinal bending and tangential extension resulted from differential transport to the foreland. It could accommodate axis rotation and strain decrease toward the foreland (after Marshak [7]); d) Finite strain in Jiaochang, showing tangential extension and strain increase toward the apex.

There are many factors affecting the deformation and displacement-path trajectories in curved fold-thrust belt. As discussed by Ries and Shackleton [26], Marshak [7] and Sussman and Weil [8], there are two types of orogen curvature: the asymmetric and symmetric types. The asymmetric orogen curvature has a simple shear along one end of the thrust belt due to movement on a lateral strike-slip fault, e.g., the Adelaide Fold-thrust Belt [27]. With the gradually increasing magnitude of stretch, it progressivly rotates to form a typically asymmetric style in magnitude and orientation of the three-dimensional finite strain. In particular, the strain and deformation increases toward the boundary strike-slip fault (*Fig. 10b*). In contrast, the symmetric orogen curvature is much more complicated. As a pure-bending orogen curvature such as the Cantabria Arc of northern Spain [28], the strain remains the same, but the orientation varies without undergoing tangential extension and vertical-axis rotation (*Fig. 10a*). In differential transport process [7], non-uniform translation results in tangential extension and different rotation of the terminus relatively to the orogen curvature (*Fig. 10c*). In general, there can be only one, or multiphase deformation of curvature-building (e.g., the Wyoming structure in the US, Weil et al. [9]) to form the primary and progressive curvature structure [8].

The three-dimensional strain data of our samples show three principal axis of strain sub-parallel to the fold axis with roughly E-W strike, being sub-perpendicular to the transport direction (N-S trending), except for samples S109-01 and S125-01 (*Fig. 6*). In particular, the intersection angle between orientation of the principal axis and bedding of sample increases from ∼30° in the north to ∼50° in the south, and even to 70° locally (*Table 1*). Based on these observations, we thus propose three possible explanations for the observed contradiction in the strain.

The first hypothesis is that the juxtaposition of coaxial pure shear and simple shear results in successive increment of strain, and similar lineation switching between the principal and other axes [29], particularly in transpressional shear zone. These successive events thus added to significant uncertainty in strain style as indicated by K values. The second hypothesis is that the reorientation of the recrystallized grains without an accompanying flow of material deformation resulted in the finite strain of three-dimensional stress tensor [30], [21]. These grains boundary migration represent recrystallization mechanism without the lateral boundary conditions. It thus results in the changing of principal axis and strain style. Alternatively, we suggest that the superposed multiphase deformation could cause localized variation in transport direction, such as the second deformation rotating the first fabric elements in the Xiaojing structure, as pointed out by Harrowfield and Wilson [3]. Therefore, superposed multiphase deformation could change the ratios of principal axis and the strain style. It is difficult to rule out any of these hypotheses based on the macro- to micro- structure alone. No single mechanism can explain with certainty all the evidence and data we have collected.

Given possible explanation for the strain in the Jiaochang, several features lead us to favor the hypothesis of progressive N-S shortening as predominantly accounting for the formation of the Jiaochang structure. These are: (a) a progressive D_1_–D_2_ deformation with insignificant post-D_2_ deformation; (b) a southward increase in deformation intensity and metamorphic condition; (c) although finite strain data shows similar magnitude in the Jiaochang (e.g., γ ≈ 1.8, c ≈ 27% and ε_s_ ≈0.9), there is much larger strain intensity values at the western limb than at the eastern limb; (d) the long-axis orientations are subparrellel to the fold axis, and roughly symmetric between two limbs; (e) no high-angle plunge and various lineation were observed in this area, which is typical feature related to transpressional movement [31]. Therefore, we concluded that the progressive N-S shortening had played an prominent role in control of the Jiaochang structure during Indosinian orogeny (*Fig. 10*). It further indicates that a simple-shear mechanism was not the predominant mechanism for structural evolution of the Jiaochang structure.

As suggested by Dirk et al. [4], Chen et al. [1] and Worley and Wilson [5], there was a sinistral transpressive shear at Wenchuan-Maowen fault at the southeast boundary of the Songpan-Ganzi fold-belt, which affected the development of curvatures in the region. Tangential extension and shearing occurring in the Jiaochang area, indicate high influence of transpressive shear at the boundary fault.

### 5.3 Structural Evolution of the Jiaochang Structure

During Late Triassic time, as result of the closure of the Paleo-Tethys Ocean, significant south-oriented compression and shortening occurred in the SGFB. The subduction occurred to the west under the Qiangtang block along western Jinshajiang-Litang suture [32]–[33], to the north under the Kunlun terrain and Qinling along its northern Anyemaqen-Mianlue suture [34], [13], and thrusting southeast-ward onlapping the Sichuan basin [1], [18]–[19]. In the Jiaochang structure, all data and structures support a southward increase in deformation intensity and metamorphism from the Triassic strata in the north to the Devonian strata in the south. Further southward, our field study indicated that Silurian strata (e.g. at Shidaguan in the southern apex) are characterized by more intensive folding and penetrative cleavage. Such increasing strain and deformation from the north to the south in different curvature structures was described by Worley and Wilson [5] and Harrowfield and Wilson [3]. Thus, we infer a similar increasing gradient of deformation and metamorphism within all curvature structures in the Songpan-Ganzi, due to a progressive south-oriented shortening.

Much of the SGFB was involved in such intensive shortening and deformation during Late Triassic time. In the Jiaochang structure, the N-S shortening and deformation during D_1_ phase has accommodated the widespread upright and isoclinal F_1_ folds. Such structure is also widespread in the SGFB, as the first episode deformation structure. The strata are intruded by undeformed, crosscutting granitic plutons emplaced during Late Triassic to Early Jurassic time [14], [25].

By progressive N-S compression during D_2_ phase, intensive deformation and metamorphism further took place to accommodate the second foliation, lineation and minerals growth in the central and southern parts of the Jiaochang structure. Harrowfield and Wilson [3] proposed that the strong south-oriented thickening and metamorphism resulted in sub-horizontal basal decollement between the sedimentary pile and the basement at the Xiaojin structures, followed by a syntectonic exhumation from a diapiric rise of its migmatised base core. Roger et al. [14] and Yan et al. [35] also described such features in the Yajiang and Jianglang structures, respectively. Thus, we suggest that a similar process took place at the Jiaochang structure, influenced by regional geological constrains.

Due to intensive deformation and metamorphism, granitic plutons being result of deeper curst melting are widespread in the SGFB [14], [36]. A large-scale decollement has been recognized [3], [25], [15], indicating a later separation of the Triassic sequence from the underlain strata, and thus a different deformation and metamorphism between them [16]–[17]. It accommodated a large-scale southward thrusting of sedimentary strata, and resulted in approximately ∼200 km shortening [13].

The intensive deformation and metamorphism could accommodate decollement and diapiric rise of the migmatized base core in the Jiaochang, resulting in a northward thrusting structure formed in the hinterland. A curvature structure of the Jiaochang was finally formed after the second episode of deformation (D_2_). It was followed by post-D_2_ deformation during Yanshanian and Himalayan epochs, with insignificant impact on the Jiaochang architecture.

Due to strong south-oriented compression, sinistral transpression synchronously occurred during the same time along the southeastern boundary of the Songpan-Ganzi area, resulting in southeast-oriented emplacement of the Longmenshan [1], [18]. An additional tangential shear resulted from sinistral transpression at the boundary fault, probably contributing to the rotation of the strain ellipses in the Jiaochang.

## Conclusions

Our study of the Jiaochang structure indicates a progressive D_1_–D_2_ deformation with insignificant post-D_2_ deformation, corresponding to two episodes of foliation, lineation and mineral growth. The deformation is characterized by nearly coaxial N-S shortening indicated by E–W striking, sub-parallel to parallel foliation (S_1_,S_2_) and lineation (L_1_,L_2_). The progressive D_1_–D_2_ structure is widespread in the southern and central region of the Jiaochang area; only D_1_ structure crops out at the north. Thus most of the structures support a southward increase in deformation intensity and metamorphism grade.

Based on the micro deformation and metamorphic structures, most of the porphyroblasts and minerals (e.g. pyrite and biotite) have two growth phases with localized growth in the third phase (muscovite). It highlights the importance of the D_1_–D_2_ deformation and metamorphism in the evolution of the Jiaochang structure, in which the pervasive foliation and multiphase mineral growth predominantly took place during the first to second episodes of the deformation (D_1_–D_2_). According to three-dimensional strain analyses, the strain intensity (γ), compression ratios (c%) and octahedral strain intensity (ε_s_), all indicate a similar feature across the Jiaochang structure (i.e., γ ≈ 1.8, c ≈ 27%, ε_s_ = 0.9). The long-axis orientations are subparrellel to the fold axis, and are roughly symmetric between two limbs in the northern region. Moreover, the Flinn values increase from ∼2 in the north to ∼6 in south. Thus, we propose that the Jiaochang structure could be interpreted by progressive N-S trending, D_1_–D_2_ coaxial shortening and deformation during the Indosinian epoch, followed by insignificant post-D_2_ deformation under N-S compression.
